# Systematic review of emerging technologies in vascularized composite allotransplantation

**DOI:** 10.3389/frtra.2026.1760147

**Published:** 2026-03-09

**Authors:** Anjali Chakradhar, Melody Yu, Simon G. Talbot

**Affiliations:** 1 Harvard Medical School, Boston, MA, United States; 2 Division of Plastic Surgery, Brigham and Women's Hospital, Boston, MA, United States

**Keywords:** digital innovation, immunosuppression innovation, technology, vascularized composite allotransplantation (VCA), virtual surgical planning

## Abstract

Vascularized composite allotransplantation (VCA) enables functional reconstruction for patients with extensive tissue defects, but has obstacles such as immune rejection, lifelong immunosuppression, and systems-level barriers that limit widespread adoption. VCA has rapidly evolved through novel surgical, immunologic, and bioengineering technologies. This systematic review synthesizes the current landscape of emerging VCA technologies, identifies literature gaps, and emphasizes opportunities for future research. We conducted a systematic literature review of PubMed and Embase with supplemental manual reference review. English-language experimental studies reporting novel VCA technologies and innovations published after 2004 were included. Case reports, reviews, studies without technological innovation, and non-English publications were excluded. Seventy-two studies fit the inclusion criteria. Novel immunomodulation strategies including belatacept, phototherapy, siRNA therapeutics, and tolerance induction via regulatory T-cells show potential to reduce systemic immunosuppression, while mesenchymal stem cell approaches may increase graft tolerance. On another front, advanced surgical techniques with real-time monitoring and nerve regeneration protocols are looking to promote functional recovery. Digital innovations like 3D modeling and virtual surgical planning allow for patient-specific preoperative planning and intra-operative assistance. In parallel, machine perfusion and cryopreservation extend tolerable ischemia times and may also enable early rejection detection. Similarly, biomarkers and imaging provide early and noninvasive rejection prediction. On a bigger scale, patient selection incorporating evidence-backed psychosocial factors and communication training work to address systems-level barriers to expand access. Ongoing research to translate these innovations into clinical practice will be important in realizing the potential of VCA.

## Introduction

Vascularized composite allotransplantation (VCA) is an advancing frontier for patients with complex reconstruction needs after significant tissue loss or irreversible damage. VCA is the transplantation of multiple tissue types in a single allograft, including skin, muscle, fat, bone, and/or nerves. It is most commonly performed with upper extremities, but also spans craniofacial, abdominal wall, lower extremities, and genitourinary systems. The goal of VCA is to restore form, function, and quality of life for patients in whom conventional reconstruction would yield suboptimal results.

Major challenges to VCA adoption include allograft rejection and lifelong systemic immunosuppression with associated toxicity as well as complex surgical and post operative care that limits accessibility.

**Figure 1:**
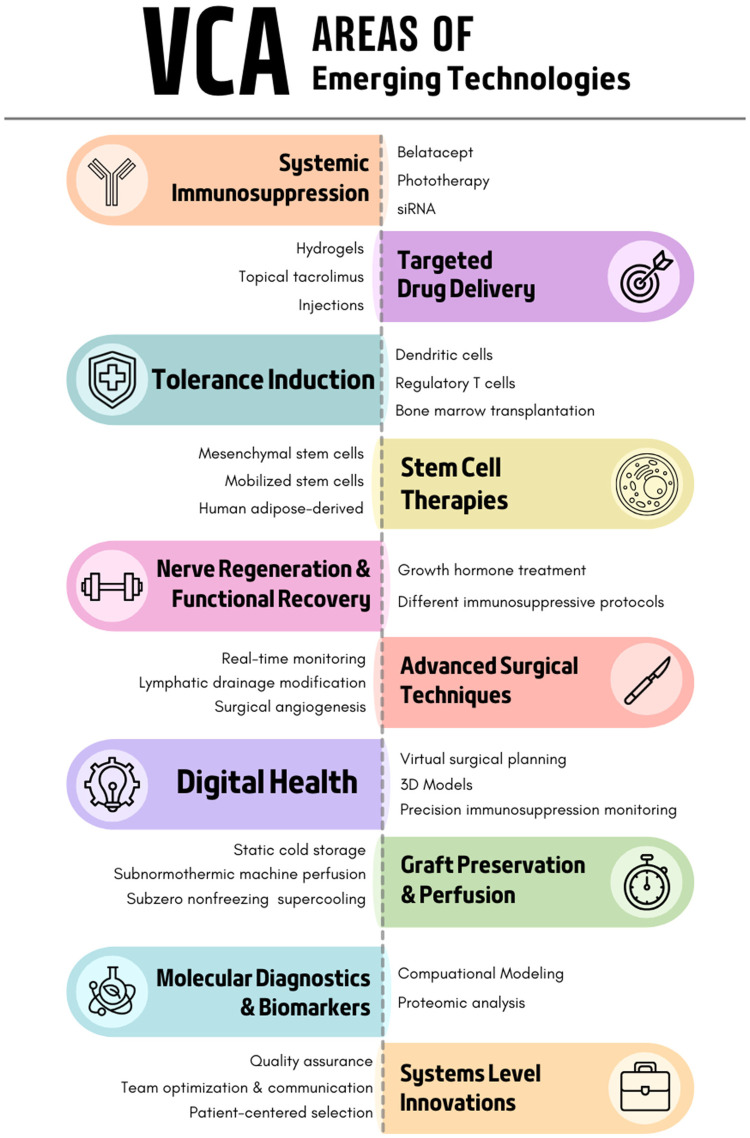
Overview of emerging technologies in VCA.

Emerging technologies across multiple domains seek to address these challenges. Current directions of research include targeted immunosuppression, tissue engineering, stem cell tolerance induction, and digital innovation. Given the rapid emergence of breakthrough technology in VCA, we systematically reviewed novel innovations across the field to characterize the current landscape. To address the breadth of technologies covered, this review functions as a hybrid systematic-scoping review. Figure 1 is a high-level summary of the innovations explored below.

## Methods

### Search strategy

We framed our research question using the Patient, Intervention, Comparison, and Outcome (PICO) framework and search strategy. For patients of vascularized composite allotransplantation (P), do emerging technologies (I) compared to standard of care approaches (C) perform better in measures such as graft survival, functional recovery, immunosuppression requirements, and quality of life outcomes (O)?

### Inclusion and exclusion criteria

English language experimental studies reporting novel VCA technologies and innovations were included. Excluded studies were non-English publications, case reports, reviews, studies without technological innovation, and articles published prior to 2004, given that the first VCA was performed in 1998.

### Study selection and data extraction

We conducted a systematic literature review to describe the novel technological innovations in VCA. We used the strategies in Supplementary Appendix 1 and 2 to search PubMed and Embase and supplemented with a manual review of references. The final search was done on December 27th, 2025. Search results were imported into a literature management library (Covidence) to remove duplicates. Of the studies yielded by the search, titles and abstracts were screened by inclusion criteria, followed by a full text screen. All manuscripts were reviewed by 2 reviewers independently to formulate a single list of articles to study, with discrepancies resolved by consensus. A formal risk of bias assessment, Supplementary Appendix 3, was performed on all studies. The SYRCLE risk of bias tool for animal studies was used for animal intervention studies, such as done in Couturier et al. (1) and the Joanna Briggs Institute critical appraisal checklist was used for all other study designs as done in James et al. (2)

## Results

### Results of the search strategy

**Figure 2:**
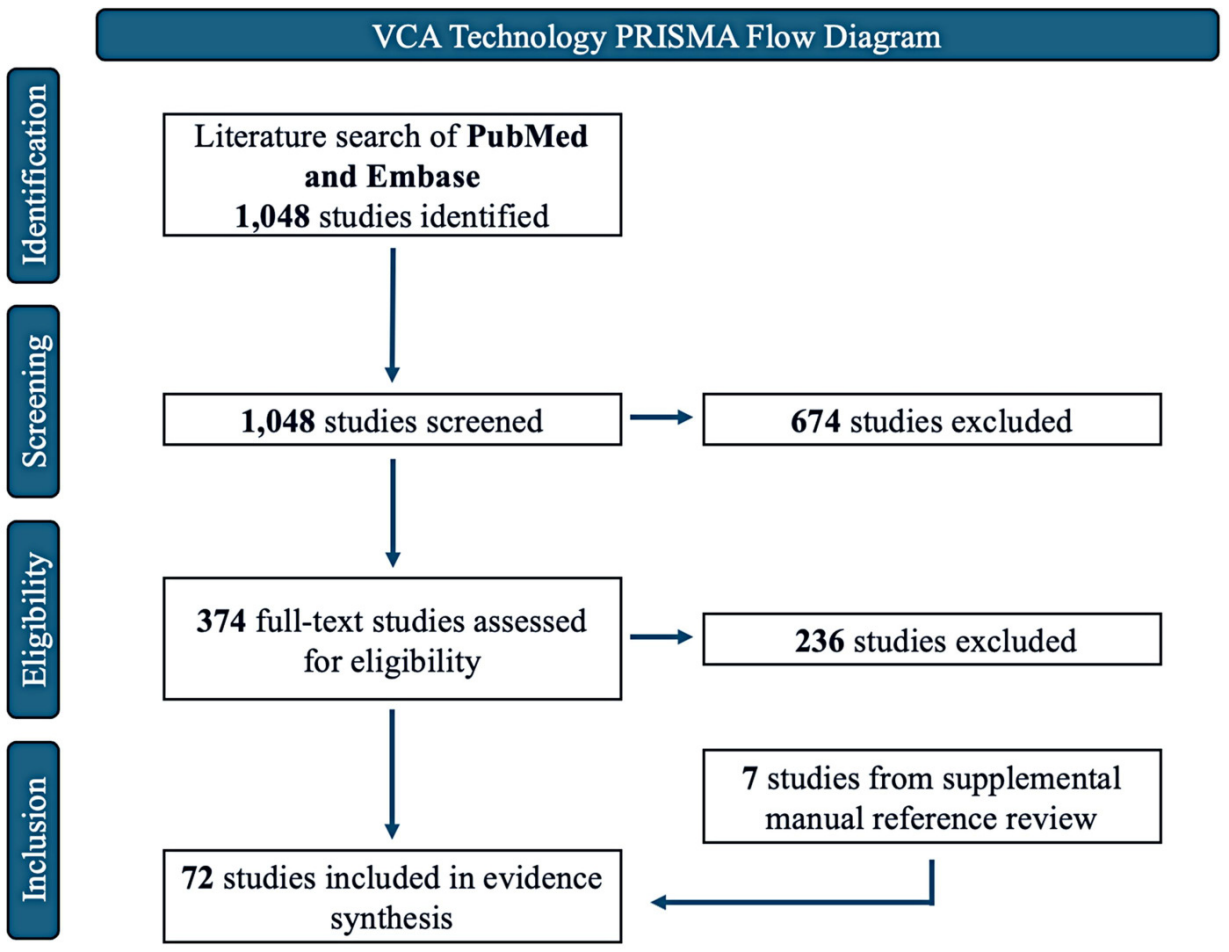
VCA technology PRISMA flow diagram.

Figure 2 provides the Preferred Reporting Items for Systematic Reviews and Meta-Analyses (PRISMA) flowchart for the study selection process. Of the 1048 studies screened in PubMed and Embase, 674 studies were excluded at the screening stage due to lack of relevance. 374 studies were then progressed to full-text review, of which 65 studies met eligibility criteria. A manual reference review from included studies revealed 7 additional studies, yielding a total of 72 studies included in the following evidence synthesis. Meta-analysis was not feasible due to heterogeneous reporting of outcomes, variable patient populations, and diverse study methods.

The results are presented below as a broad overview of emerging technologies currently under investigation in the field of VCA. Key takeaways are presented from each study, along with an assessment of the potential risk of bias. Results are organized in a combined chronological and conceptual sequence to reflect the progression of technological ideas over the past 20 years. We have concluded the paper with a synthesized discussion that identifies overarching trends in the literature highlighting opportunities for future research to advance the field in clinically impactful directions.

### Immunosuppression

Systemic immunosuppression required for VCA carries important risks including infection, metabolic disturbances, and nephrotoxicity. Extensive innovation focuses on developing less toxic or reduced systemic immunosuppression, developing targeted drug delivery systems, and tolerance induction. See Table 1 for an overview of the studies on immunosuppression that will be discussed below.

**Table 1:** Immunosuppression.

Author	Year	Model	Intervention	Results
*Systemic Immunosuppression*				
Grahammer et al.	2017	Clinical case series: 4 hand transplants	Belatacept	Well-tolerated and maintained graft survival in 3 of 4 patients
Hui-Chou et al.	2012	Rat abdominal wall	Doxorubicin	↑ 86% graft survival time than untreated
Liu et al.	2024	Rat hindlimb	Phototherapy with short-term antilymphocyte serum+cyclosporine A	↑ VCA survival: ↑ Tregs and TGF-*β*1, ↓ IL-1β
Chang et al.	2009	Rat leukemia cell line; alloskin and CTA model	siRNA	↓ JAK3 *in vivo* and *in vitro*; ↓ inflammatory infiltration
*Targeted Drug Delivery*				
Gharb et al.	2013	Rat hemiface	Topical tacrolimus and clobetasol	Both treated acute rejection; topical tacrolimus after systemic immunosuppression prevented complications
Fries et al.	2019	Porcine orthotopic forelimb	Graft-implanted tacrolimus-eluting hydrogel system	Low dose prolonged survival (56–93d); high dose not tolerated (24–42d)
Feturi et al.	2022	Rat hindlimb	Tacrolimus-eluting polycaprolactone disk implantation	Long-term allograft survival (>200d) with low systemic tacrolimus levels; high drug concentrations in allograft
Feturi et al.	2024	Rat hindlimb	Topical tacrolimus+mycophenolic acid	>100d VCA survival; high local drug concentrations; normal renal function and ↓ 90% systemic tacrolimus
Chen et al.	2020	Clinical case series: 2 hand transplants	Topical tacrolimus and clobetasol	Resolved acute rejection by decreasing perivascular lymphocyte infiltration, ↓ CD3+ T-cells and CD20+ B-cells, ↑ FoxP3+ Tregs
Gama et al.	2020	Macaque radial forearm flap	Tacrolimus TyroSphere gel dressing	Enhanced skin penetration while preventing systemic absorption but failed to prevent VCA rejection
Wang et al.	2020	Rat hindlimb	PLGA microsphere injection	↑ VCA survival >150d vs. 34.5d with oral therapy
Olariu et al.	2017	Rat hindlimb	Single intra-graft tacrolimus injection	↑ VCA survival, avg 70.5d; rejection-free >200d
Arenas Hoyos et al.	2024	Porcine hindlimb	Tacrolimus glycerol monostearate hydrogel injection	↓ 62%–84% neutrophil extracellular traps; repeated use ↑ VCA survival and ↓ systemic toxicity
Lee et al.	2025	Rat hindlimb; Porcine gracilis flap	PLGA depot injection (tacrolimus+rapamycin)	Controlled release with reversibility; VCA survival >90d
Sutter et al.	2019	Rat hindlimb	Rapamycin-loaded *in situ* forming implants	↑ VCA survival; ↑ Tregs with subtherapeutic systemic levels

### Systemic immunosuppression strategies

In 2017, researchers adapted belatacept, a cytotoxic T-lymphocyte-associated protein used in kidney transplantation, as a calcineurin inhibitor-sparing strategy in four hand transplant recipients (3). Belatacept maintained graft survival in three of the four hand transplant recipients. One patient with pre-existing high donor-specific antibodies experienced rejection requiring amputation, though the relationship to belatacept remains unclear. Belatacept is now seeing increased usage in VAC recipients.

A 2012 *in vivo* study tried a novel approach of repurposing doxorubicin chemotherapy's immunosuppressive side effects for VCA. They found an 86% increase in graft survival time compared to untreated rats, probing an alternative strategy for patients who may develop a malignancy after VCA (4).

A 2024 *in vivo* rat study demonstrated that phototherapy combined with short-term antilymphocyte serum and cyclosporine A significantly prolonged VCA survival by increasing regulatory T cells (Tregs) and TGF-*β*1 while decreasing IL-1β, offering non-pharmacological adjunct therapy to reduce long-term immunosuppression requirements (5).

Chang et al. investigated siRNA as an immunosuppressive approach, demonstrating effective JAK3 downregulation both *in vivo* and *in vitro* for the first time and reduced inflammatory infiltration in rat VCA grafts compared to controls. This indicates siRNA's potential for precise immunosuppression with fewer systemic side effects (6).

In a different approach, bioengineered tissues are being studied to limit immunosuppression requirements. Researchers at the University of Toronto created tissue-engineered scaffolds by decellularizing donor tissues, and recellularizing with cells derived from the recipient (7). Current limitations to this approach include cell selection and function of elements such as vasculature and muscle.

### Targeted drug delivery

Active investigation is underway into targeted drug delivery systems to reduce systemic immunosuppression requirements and associated toxicities, while still effectively preventing rejection.

In 2013, researchers compared the efficacy of topical tacrolimus vs. clobetasol with and without systemic immunosuppression in 76 rat hemiface allotransplants (8). Topical use of both agents was shown to effectively treat episodes of acute rejection. Topical tacrolimus after systemic immunosuppression performed best in rejection prevention, with clobetasol having associated lymphocyte depletion and tissue atrophy.

In 2019, researchers engineered a tacrolimus-eluting hydrogel as a local, implantable drug delivery system that limits systemic drug toxicity in VCA patients (9). In a porcine orthotopic forelimb VCA model, low dose tacrolimus hydrogel treatment (49 mg) showed long-term graft survival to 56–93 days, while high dose tacrolimus hydrogel treatment (91 mg) showed survival to 24–42 days but was not well tolerated. This graft-implanted TAC-eluting hydrogel system has potential to be a clinically translatable implantable drug delivery system that achieves VCA graft survival and reduces systemic immunosuppressive drug toxicity.

In 2022, Feturi et al. created tacrolimus-eluting polycaprolactone disks and implanted them into transplanted rat hindlimbs for site-specific immunosuppression. This intervention achieved long-term allograft survival (>200 days) with low systemic tacrolimus levels (2–5 ng/mL), high drug concentrations within the allograft, without signs of metabolic and infectious complications (10).

In 2024, the group built on this work, evaluating if topical application of tacrolimus and mycophenolic acid to transplanted rat hindlimbs, combined with low dose systemic tacrolimus (0.1 mg/kg/day), could improve allograft survival while reducing systemic exposure. Topical tacrolimus plus mycophenolic acid achieved >100 day VCA survival, maintaining high local drug concentrations while preserving normal renal function and avoiding systemic toxicity at a 90% reduction in systemic tacrolimus dose compared to controls (11). In two human hand transplants, topical tacrolimus and clobetasol successfully resolved acute rejection by decreasing perivascular lymphocyte infiltration, suppressing CD3+ T cells and CD20+ B cells while increasing FoxP3+ Tregs, thus offering adjunctive therapy without pulse-steroid requirements (12).

However, topical tacrolimus therapies are at times limited by poor skin penetration, warranting other methods of tacrolimus delivery. To address this, a 2020 study engineered tacrolimus TyroSphere gel dressing containing nanoparticles that enhances skin penetration while preventing systemic absorption (13). Despite the improved delivery system, VCA rejection still occurred in this system due to insufficient local immunosuppression to replace systemic therapy.

Other methods of providing sustained immunosuppression also show promise. In a 2020 rat hind limb model, a single injection of polylactic-co-glycolic acid microspheres loaded with tacrolimus, mycophenolate mofetil, and prednisolone achieved median graft survival exceeding 150 days, compared to 34.5 days with standard daily oral therapy with the same triple regimen (14). The sustained-release microsphere system maintained higher and more stable plasma and tissue drug concentrations, prolonging immunosuppression and reducing rejection, while standard oral therapy was limited by suboptimal pharmacokinetics and fluctuating drug levels in the model. This approach demonstrates the potential for single-injection, sustained-release immunosuppression to extend VCA survival, with clinical implications of improved patient compliance and reduced administration burden compared to standard oral therapy (14).

Similarly, a 2017 *in vivo* study in rodents showed a single intra-graft tacrolimus injection significantly prolonged VCA survival, with an average of 70.5 days and remaining rejection-free beyond 200 days, hence achieving tissue-specific immunosuppression without systemic toxicity (15).

In 2024, Arenas Hoyos et al. developed a new local drug delivery system consisting of a triglycerol monostearate hydrogel loaded with tacrolimus and injected directly into grafts in a multiple MHC-mismatched porcine VCA model. *In vitro*, tacrolimus treatment of stimulated neutrophils had a 62%–84% reduction in neutrophil extracellular traps, a marker of innate immune involvement. Repeated intra-graft administration significantly prolonged graft survival and minimized systemic toxicity (16).

In a 2025 study, researchers created an injectable polylactic-co-glycolic acid depot that releases tacrolimus and rapamycin within the graft (17). A limitation to local immunosuppression delivery systems is burst release, which is an initial release of drug when the delivery system is first implanted that can cause graft toxicity. The injectable polylactic-co-glycolic acid depot suppressed burst release by incorporating small molecule drug binding agents. They also demonstrated reversibility, as removal of the depot triggered a rapid decline in tacrolimus levels. In a fully MHC-mismatched porcine model of VCA, their delivery system maintained therapeutic drug levels and extended graft survival beyond 90 days with minimal rejection.

Besides tacrolimus, other drugs such as rapamycin also prove effective in targeted drug delivery systems. Sustained low-dose rapamycin delivery via rapamycin-loaded *in situ* forming implants were developed in a 2019 study that significantly prolonged VCA survival while expanding T regulatory cells and achieving subtherapeutic systemic levels, also potentially improving patient compliance (18).

### Tolerance induction

Tolerance induction is an area of research in VCA that aims to eliminate chronic systemic immunosuppression and its associated toxicities by creating a state in which the recipient's immune system accepts the transplanted allograft as “self”. This work draws on the success of renal transplant tolerance in the setting of ablative stem cell transplants in living donor settings, enabling immunosuppression withdrawal. However, these protocols require lengthy preconditioning which is impractical with a deceased donor and have toxicities such as infection, organ dysfunction, and graft-vs.-host disease that may not be survivable when combined with major VCA operations.

A 2011 study showed that compared to tacrolimus, cyclosporine had a higher proportion of recipients achieve long-term (150 day) survival of the allograft and higher chimerism levels for VCA tolerance protocols, demonstrating tolerance induction with less myeloablative conditioning (19). This provides evidence for optimizing immunosuppressive drug choice and reducing conditioning intensity while maintaining efficacy in VCA. Furthermore, a 2013 study demonstrated that, compared to full-thickness skin transplantation models, MHC mismatched mice were susceptible to tolerance induction via CD4(-) CD8(-) double negative Treg-based therapy combined with anti-lymphocyte serum, rapamycin, and IL-2/Fc fusion protein, resulting in donor-specific tolerance with macrochimerism and increased CD4+ Foxp3+ Tregs that was uniquely successful in VCA but not in skin grafts (20). A subsequent 2015 rat hindlimb transplant study found that human IL-2 fusion protein (hIL-2/Fc) in combination with antilymphocyte serum and short-term cyclosporine promoted Treg proliferation, suppressed effector T-cells, and induced VCA tolerance with long-term graft survival (21). Two types of acute rejection, progressive and reversible, were also identified in treated recipients together with differential gene expression profiles, with reversible rejection showing higher Treg to Teff gene expression.

Cellular immunomodulatory therapies, such as with dendritic cells, are interesting approaches to induce VCA tolerance by increasing Treg activity and suppressing effector T-cell responses. In a 2007 rat hindlimb VCA model, IV injection of recipient bone marrow-derived dendritic cells pulsed with donor allopeptide was found to prolong VCA survival from 5 to 8 days while reducing donor T-cell infiltration (22). This demonstrates a potential for donor-specific tolerance induction through antigen-presenting cell manipulation. Similarly, donor alloantigen-pulsed recipient immature dendritic cell therapy with transient immunosuppression was shown in a 2009 study to achieve over 200 day VCA survival through donor-specific T cell hyporesponsiveness and increased CD4+/CD25 + and CD4+/FoxP3+ Tregs, thus establishing an *ex vivo*-educated dendritic cell approach for tolerance induction that reduces long-term immunosuppression requirements (23). A 2016 study in mice demonstrated that TIM-3-modified (T cell Ig domain and mucin domain 3) mature dendritic cells significantly prolong VCA survival by inducing CD4+ T cell differentiation into Tregs, facilitating lymphocyte apoptosis, and decreasing proliferation, ultimately offering a targeted immunomodulation approach that rebalances effector and regulatory immune responses (24).

In 2020, a preclinical experimental study mimicked how the human body naturally recruits Tregs in a synthetic drug delivery system aimed at inducing tolerance in rat hindlimb VCA models (25). A microparticle system was engineered to release the Treg-recruiting chemokine CCL22, termed “Recruitment-MPs”, that prolonged hindlimb allograft survival indefinitely (> 200 days) and promoted donor-specific tolerance that was demonstrated by the acceptance of secondary donor skin grafts and rejection of third-party skin grafts. The Recruitment MPs successfully recruited Tregs into the allograft and enhanced Treg function without affecting the proliferation of conventional T cells. Synthetic CCL22 was then tested *in-vitro* showing preferential migration of human Tregs, suggesting a potential for translation into human VCA systems (25). However, further validation in large animal studies is currently lacking.

Other cellular therapies beyond dendritic cells have also proved promising. A 2012 rat hindlimb study introduced donor-derived transplant acceptance-inducing cells (TAICs), which are deactivated immunoregulatory macrophages. These TAICs significantly delayed VCA rejection from 5.6 to 7.7 days through adoptive transfer of *ex vivo* cultured tolerance-inducing cells, establishing a novel macrophage-based immunomodulatory approach (26). A 2020 rat hindlimb VCA study demonstrated that adipose-derived stromal cells combined with a radiation-free conditioning protocol achieved 86% long-term VCA survival without chronic immunosuppression through persistent mixed chimerism and elevated Tregs, providing a clinically translatable tolerance induction strategy (27). A 2021 rat study created donor-recipient chimeric cells (DRCC) by fusing donor and recipient bone marrow cells using polyethylene glycol (28). DRCC therapy was successful in inducing multilineage chimerism and also extended VCA survival to 79 days. In 2022, Siemionow et al. created a novel human hematopoietic chimeric cell line through fusion of CD34 + cells from two unrelated bone marrow donors (29). *in-vitro* evaluation showed increased expression of Tregs and a 40-fold increase of the protolerogenic cytokine IL-10 at 21 days after fusion (*P* < 0.0001).

Bone marrow transplantation incorporation provides advances in VCA protocols. A 2013 rat VCA study achieved 80% graft acceptance with 300 cGy total body irradiation through simultaneous bone marrow transplant with non-myeloablative conditioning via Treg-mediated suppression and thymic deletion, addressing deceased donor time constraints (30). More recently, a 2022 rat VCA study demonstrated that inclusion of vascularized bone marrow (VBM) significantly improved mystacial pad allograft survival (>90 days vs. 70 days) through persistent chimerism and donor-specific T cell hyporesponsiveness vs. non-VBM transplants. They demonstrated VBM as an efficient strategy for inducing tolerance in osseous-free allografts without lifelong immunosuppression (31).

The success of these tolerance strategies in human clinical trials has not yet been evaluated.

### Stem cell therapies

Mesenchymal stem cells (MSCs) can enhance graft tolerance, reduce reliance on systemic immunosuppression, and mitigate graft-vs.-host disease. A 2010 rat hindlimb study demonstrated that MSC co-infusion with bone marrow transplantation achieved stable chimerism in 14/15 recipients (vs. 2/7 without MSCs) while preventing graft-vs.-host disease, providing a safer approach for chimerism-based tolerance induction (32). However, these findings are limited by the toxic conditioning protocols required, including antilymphocyte serum, rapamycin: 0.2 mg/kg/d; days 0 approximately 130, and 3 Gy total body irradiation. A 2017 study in rat VCA models demonstrated that repetitive adipose-derived MSC therapy on POD 4, 8, and 15 achieved 50% long-term VCA acceptance with enhanced chimerism (12.9%), increased regulatory T-cells, and reduced arterial intimal thickness, establishing optimal dosing protocols for MSC-based immunomodulation (33).

MSCs moreover have the potential to reduce immunosuppression. A 2018 rat hindlimb study introduced *ex vivo* graft engineering that delivers MSCs directly to allografts before transplantation, achieving localized immunomodulation with prolonged rejection-free survival to potentially reduce systemic immunosuppression burden (34). A 2021 study established a large-scale cryopreserved bone marrow bank from deceased organ donor vertebral bodies with matched MSCs, providing >1,000-fold higher MSC yields than living donor aspirates. This innovation addresses previous limitations in cell yield (35).

Other stem cells also have been proven to be effective towards enhanced VCA survival and tolerance induction. A 2016 haploidentical canine study comparing bone marrow vs. G-CSF mobilized peripheral blood stem cells for simultaneous hematopoietic cell and VCA transplantation found mobilized stem cells achieved superior tolerance (100% vs. 25% acceptance) with long-term survival over 90 weeks. This highlights mobilized stem cells as an optimized source for tolerance induction despite increased graft-vs.-host disease risk (36).

Furthermore, a 2019 study demonstrated that human adipose-derived stem cells (hASCs) genetically engineered with chemokine receptor 7 (CCR7) receptor for targeted migration to secondary lymphoid organs in rat VCA model were able to significantly prolong VCA survival through enhanced immunomodulation at sites of immune initiation while regulating Th1/Th2 and Tregs/Th17 balance compared to unmodified hASCs (37).

### Nerve regeneration and functional recovery

Recent therapeutic and immunosuppressive innovations have demonstrated increased effectiveness of nerve regeneration and functional recovery in VCA models.

A 2024 rat orthotopic forelimb model explored nerve regeneration with functional recovery when treated with porcine-derived growth hormone (GH) (38). GH-treated rats showed improved neuromuscular junction reinnervation and grip strength recovery without increasing rejection risk, with no deficits in muscle atrophy, myelination, axon diameter, or axon counts.

Furthermore, a 2018 rat sciatic nerve VCA model evaluated motor function recovery under different immunosuppressive protocols (39). The researchers compared no treatment, 2 mg/kg tacrolimus monotherapy, 1 mg/kg tacrolimus+15 mg/kg mycophenolate mofetil (MMF), and a high-dose triple therapy protocol of 2 mg/kg tacrolimus+30 mg/kg MMF and prednisone. While all immunosuppressive regimens significantly improved motor recovery compared to controls, the triple therapy enhanced ankle contracture and had superior electrophysiology outcomes.

### Advanced surgical techniques

Novel surgical techniques have significantly advanced VCA.

A 2018 study introduced an improved non-suture cuff technique using the abdominal aorta for end-to-end anastomosis along with laser speckle imaging for intraoperative detection of vascular complications (40). This technique improved preclinical VCA model reliability and reduced surgery time by 58% and achieved 90% success rates.

A 2024 study combined vascularized allogeneic spinal cord transplantation with polyethylene glycol fusion in canines, expanding VCA beyond traditional composite tissues by establishing spinal cord as a transplantable vascularized organ and demonstrating further evidence for the use of polyethylene glycol in nerve fusion (41). Polyethylene glycol fusion is believed to promote immediate axonal continuity by fusing severed nerve membranes, bypassing Wallerian degeneration and accelerating functional recovery. This may have applications in VCA outside of the spine as well.

A 2018 rat study using an annular plastic holder to minimize allograft contact and inhibit lymphatic drainage prolonged allograft survival, reduced serum IL-2, and decreased T cell populations in draining lymph nodes, establishing the lymphatic system as a novel therapeutic target for VCA rejection prevention (42).

A 2011 study created a method for surgical angiogenesis using autogenous neoangiogenic circulation via saphenous artery-vein bundle implantation. This enabled long-term VCA survival, achieving highest cortical bone flow (4.6 mL/min/100 g) and new bone formation (6.4 μm^3^/μm^2^/yr) while reducing a need for chronic immunosuppression (43). There is also innovation in intraoperative graft perfusion monitoring. Nguyen et al. designed a real time monitoring system with near infrared fluorescence angiography to detect arterial and venous phases, quickly find perforator location, and check flap perfusion in a large animal porcine model of the hemiface (44).

### Digital innovation

Surgeons outside of VCA use models to visualize 3D space and practice surgical approaches, which improve surgical accuracy and reduce time in the operating room (45–47). This direction of innovation has potential to advance VCA, especially in craniofacial transplantation where even small deviations can result in misalignment. Guides also allow for the independent operation of donor and recipient teams through reciprocal cutting and fit systems. Table 2 is an overview of the studies discussed below.

**Table 2:** Digital innovation.

Author	Year	Intervention	Results
Cornelius et al.	2016	VSP: cutting guides for lateral scapular border flaps	VSP of bone flaps from lateral scapular border can be transferred into surgery
Antony et al.	2011	VSP	↑Accuracy in craniofacial procedures
Ciocca et al.	2012	VSP: cutting guides and reconstructive titanium bone plates for osteotomies	No postoperative complications; good facial soft-tissue contour; accurate resection boundaries
Jacobs et al.	2013	VSP: patient-specific CAD/CAM cutting and fixation guides	Optimize donor-recipient craniofacial bony matching
Vyas et al.	2022	VSP+3D-printed guides	Improved osteotomy placement and alignment of transplanted tissue
Soga et al.	2011	VSP: 4D (3D space+time) non-invasive vascular imaging	Quantified external carotid circulation time; detected target vessels and differentiated arteries/veins; computed tissue perfusion; CT > MRI for small vessels and artifacts, both gave arterial/venous roadmaps
Cho et al.	2021	VSP: mixed reality models as visual adjuncts in craniofacial VCA	Fast, low-cost holograms for rehearsal and visualization
Murphy et al.	2015	Intraoperative “real-time cephalometry”	Small deviations; real-time cephalometric feedback; improved donor positioning
Shah et al.	2024	Digital workflow for cross-gender facial transplantation using virtual models of donor and recipient craniofacial anatomy and patient-customized cutting guides	Precise osteotomy simulation; ↓OR time by 4.5 h; cephalometric measurements within norms. Enables pre-operative virtual optimization of allograft design and screening for anatomical disharmony in cross-gender donor-recipient pairs before surgical commitment
Lantigua et al.	2021	Sensitive, low-cost, portable ELISA device for point of care tacrolimus detection	Finger prick; <10 min results with 50 μl of blood
Charlès et al.	2023	Point of care device using gold nanoparticles that monitors salivary tacrolimus within 15 min	Results limited; salivary tacrolimus monitoring is still in early stages

Guides created from virtual surgical planning (VSP) were demonstrated to be more accurate than conventional techniques across craniofacial procedures, including mandibular reconstruction with free fibula, iliac flaps, and lateral scapular border transfers (46). In 2013, a study investigated a customized VSP for cutting and fixation guides in craniofacial VCA to improve donor–recipient bony matching (48). They were able to match 3D skeletal characteristics for proper bony contact and dental occlusion, with a goal to achieve a “snap-fit” inset intraoperatively. Initial results were positive using this method in a cadaveric model that was postoperatively verified through overlay analysis.

Researchers at Mayo clinic have also been developing VSP and 3D-printed guides for use in craniofacial VCA that allow them to optimize osteotomy placement and alignment of transplanted tissue (49).

Digital innovations are helping surgeons identify recipient and donor vessels for anastomosis in pre-operative planning. In 2011, VCA surgeons collaborated with radiologists to generate a four-dimensional (3D space plus time) non-invasive vascular imaging technique using contrast-enhanced CT angiography and MR angiography and post-processing (50). They were able to quantify rapid external carotid circulation times, tell arteries apart from veins, and compute tissue perfusion for face transplantation candidates. This was possible despite the varied indications for procedure (gunshot wound, high voltage injury, burn). Their novel technology reliably detected target vessels, with CT outperforming MRI for small vessels and in patients with metal artifact, while both provided phase-separated vascular maps to guide surgical planning.

Researchers at the Cleveland Clinic have investigated mixed reality models to assist with visualization in facial transplantation (51). This approach was both preoperative and intraoperative. It created interactive holographic models that allowed for preoperative rehearsal and even visualization within the operative field itself. Benefits also included reduced cost and shorter fabrication time compared to 3D models. Currently, mixed reality models complement traditional VSP with 3D cutting guides, and more work is needed to compare these approaches to establish clinical outcome gains in VCA.

Real time virtual systems are also being employed intra-operatively in craniofacial transplantation. In 2015, researchers engineered and tested an intraoperative “real-time cephalometry” to address malposition-driven problems in transplantation (52). The intraoperative system continuously tracked the donor maxillomandibular segment against preidentified landmarks and updated key hybrid occlusal metrics in real time. Because the same landmarks and coordinate system were used for planning, intraoperative tracking, and postoperative assessment, the measurements during surgery and the final postoperative measurements were comparable. This approach had small deviations from the original plan (≈1–3 mm, 0.5–3°) that were within accepted orthognathic tolerances, indicating that the real-time metrics reflected final alignment with clinically useful fidelity. Real-time cephalometry provides actionable feedback to adjust the position of donor segments and assess hybrid occlusion in the OR. Given the high rates of complications reported across facial transplants (53), this approach may help improve both functional and aesthetic outcomes.

To address limited donor pools and long waitlist times, researchers created a novel digital workflow to improve the feasibility of facial transplantation across sex (54). Barriers to this include sex-specific measurements, skin texture, and social concerns. The team used CT segmentation and 3D reconstruction to create virtual models of both donor and recipient anatomy that allowed for precise planning and osteotomy simulation. They manufactured customized cutting guides that translated these virtual plans into physical templates for precise cuts to bone during surgery. It was then implemented in 2 cross-sex (male to female and female to male) cadaveric Le Fort III double jaw allotransplantations. Results showed cephalometric measurements relative to Eastman Normal Values that were consistent with established procedures within the same sex. Furthermore, this novel digital workflow reduced operative time by 4.5 h while also enabling virtual assessment of allograft design and screening for site of anatomic disharmony preoperatively.

Digital innovation is also being used in VCA to monitor drug levels in immunosuppression regimens. To monitor tacrolimus, a key element in VCA immunosuppression, trough levels are traditionally measured through serial blood draws and laboratory testing. In 2021, researchers created a low cost and portable device for tacrolimus detection at the point of care (55). It is a paper-based microfluidic device that conducts an ELISA to detect tacrolimus in small volumes of blood within 10 min. It only requires 50 μl of blood which can be obtained from a finger prick. This could represent a simpler and quicker alternative to current immunoassay-based techniques. In 2023, researchers created a point of care device using gold nanoparticles to extract and quantify tacrolimus levels in saliva within 15 min (56). However, in a retrospective analysis of 62 samples from 31 human patients of VCA or solid organ transplantation, results were limited. Salivary and serum tacrolimus levels were weakly correlated (r = 0.39) with an absolute error of 1.8 ng/mL which is substantial given tacrolimus's already narrow therapeutic range. No further published studies have built on these results.

### Graft preservation and perfusion

Prolonged ischemia is associated with graft rejection, functional deficits in VCA, and even amputation, demonstrating the need for techniques in graft preservation and perfusion (57). Static cold storage (SCS) has been the gold standard as it reduces metabolic demand and also microvascular permeability, which combats ischemic damage and reperfusion injury (58).

In a study with five human limbs, Werner et al. showed that prolonged ex situ perfusion may be feasible (59). The graft was preserved and showed no signs of myocyte damage for 24 h. This was an early investigation into the potential of ex situ perfusion for issues that arise in the clinical practice of VCA, such as long-distance allocation and complex OR coordination.

Goutard et al. created a method in 2023 for subnormothermic machine perfusion (SNMP) after SCS. It extended preservation time and mitigated cold ischemia-induced injury in *ex vivo* rat VCA (60). 3 h of recovery with SNMP following 12 h of SCS was shown to bring arterial vascular resistance, lactate, and potassium to fresh limb values. In 2024, a large animal swine model was used to compare continuous 24 h of SNMP vs. SCS (61). They found improved overall survival and normal muscle architecture with SNMP, while degeneration was seen with SCS alone 2 weeks after transplantation.

Another research avenue explored subzero nonfreezing (SZNF) supercooling combined with monitored perfusion. In 2024, researchers developed a SZNF toolkit for VCA preservation that used cryoprotectants with thermal monitoring that significantly improved arterial resistance and weight outcomes compared to SCS *ex vivo* in rats (62). The same group later used this protocol *in vivo* in rodent hindlimbs, comparing 48 h of SCS to SZNF at −4 °C (63). SZNF-preserved limbs survived 28 days while SCS limbs had early failure in 4 days with static cold storage.

### Biomarkers

Tissue-specific biomarkers allow for precision immunomonitoring and early intervention protocols aim to predict transplant rejection before histologic injury.

Computational modeling in a 2014 study was applied to a rat orthotopic hindlimb model to identify biomarkers and tissue-specific rejection patterns for treatment of acute skin rejection, a major cause of VCA failure (64). Using machine learning models, the team was able to identify cytokine biomarkers (IL-4, TNF-α, IL-12p70 for skin; TNF-α, IL-12p70 for muscle) as early predictors of rejection ahead of histological changes. They then employed principal component analysis that specified IL-1*α*, IL-18, IL-1β, and IL-4 as key drivers of rejection, showing that computational modeling may assist in detecting VCA rejection before histologic signs of damage.

In the same year, a proteomic analysis identified protein biomarkers of tolerance in a rat hindlimb model that was treated with adipose-derived stem cells and short-term immunosuppression (65). Using advanced biochemical techniques, the study identified five protein biomarkers of tolerance: increased *β*2-glycoprotein, *α*1-macroglobulin, rat-albumin, with particular emphasis on increased vitamin D-binding protein and decreased haptoglobin.

In 2015, researchers developed a noninvasive method for real-time monitoring of rejection in VCA using reﬂectance confocal microscopy (RCM) (66). RCM is a noninvasive imaging technique used in dermatology that employs lasers to create a high-resolution, magnified image of skin microstructure. In 17 specimens from 5 groin flap allotransplants of rats, total RCM score had a strong and statistically significant correlation with Banff histopathological analysis (correlation coefﬁcient = 0.653, *p* = 0.01). This could facilitate the creation of a predictive tool to predict VCA rejection in the skin without the need for invasive biopsy, processing time, and pathology assessment.

Skin biopsies are the gold standard of assessing rejection in VCA due to ease of access but do not reflect rejection in deeper tissues such as sinonasal mucosa in facial transplants. In a 2021 retrospective observational cohort study, Kauke et al. used image segmentation of CT scans to quantify maxillary sinus aeration coefficients in 6 human face transplant recipients (67). Aeration coefficients were found to be significantly reduced in cases of biopsy-proven allograft rejection, likely a reflection of underlying mucosal swelling. In this way, the authors created a novel and noninvasive radiologic marker of rejection that could address the limitations of skin biopsy in deeper tissues, facilitate earlier detection of rejection, and even help in designing individualized treatment strategies through dynamic monitoring. The authors built on this work in a 2023 retrospective cohort study of 8 facial VCA recipients where they identified another noninvasive biomarker of rejection, this time through serum laboratory measurements (68). They identified a statistically significant correlation between Banff grade of rejection and both relative basophil count and CRP, positing CRP and basophil count as potential adjunct markers of VCA rejection.

### Systems-level innovations

VCA relies on complex interdisciplinary collaboration, team dynamics, and ethical/regulatory measures, representing an active area of investigation.

### Quality assurance

In 2010, researchers created a set of guidelines for program establishment, approval logistics, and donor engagement to guide the development of site-specific protocols in VCA (69). A 2025 qualitative study then introduced a novel set of patient-reported outcome measures for upper extremity VCA (70). This facilitates a more standardized reporting of patient satisfaction, which is a current limitation to cohesive quality improvement efforts in the field.

### Team dynamics

In addition to quality assurance, systems research has also focused on improving coordination of care in VCA.

In 2017, Sweeney et al. created a clinical protocol to assist with perioperative nursing care for face transplant donors and recipients that standardized nursing workflows (71). Mayo Clinic researchers in 2022 looked to team science to create a framework with auditable routines including dedicated pre and post briefing, clear assignment of roles, and pathways for escalating issues to improve team performance (72). This innovation in team coordination is reflective of the coordinated care that successful VCA necessitates.

In 2024, researchers acknowledged the broad multidisciplinary scope of VCA and highlighted the role of donation professionals in approaching family decision makers regarding the donation of uncommon anatomical gifts, such as the face and hands, in VCA (73). They created an online system for donation professionals aimed at improving communication within VCA donation systems. Users rated it highly for effectiveness and accessibility. They showed how Learning Management Systems can meet performance metrics for organ procurement set by the Centers for Medicare and Medicaid Services to standardize training in the larger donation network. This finding demonstrates that scalable digital training systems can be developed in accordance with established insurance standards, with the potential to improve cohesiveness.

Together, these innovations focus on the multidisciplinary teamwork central to VCA, develop novel protocols to improve both individual role performance and interprofessional collaboration based on team science metrics, and show that scalable digital training systems can be implemented in accordance with established insurance standards.

### Psychosocial factors

Literature suggests that anxiety, PTSD, unrealistic expectations, and poor family support are associated with transplant loss in upper extremity VCA (74). Perceived success in upper extremity VCA is associated with strong social support and realistic expectations (75). It is important to screen for psychosocial factors and set realistic expectations for improved VCA outcomes. A 2020-22 series of studies created new evaluation protocols for transplant recipients, integrating psychosocial elements like coping skills, positive attitudes, and caregiver stability to improve transplant outcomes (76). A 2023 study by Tyner at al. developed a framework of eight patient-reported outcome domains in upper extremity VCA, addressing other quality-of-life measures such as integration/assimilation and satisfaction with sensation currently not included in standard measures (77). This blueprint is an early step toward incorporating data on psychosocial predictors in VCA.

## Discussion

Technological innovation in VCA spans immunosuppression, tolerance induction, stem cell therapy, nerve regeneration, advanced surgical techniques, digital innovation, graft preservation and perfusion, biomarkers, and systems work to improve outcomes.

Given the nature of VCA as a life-enhancing rather than life-saving procedure, its risk-benefit considerations are different from those in solid organ transplantation. Therefore, the adverse effects of chronic immunosuppression such as infection, malignancy, and nephrotoxicity become less easily justifiable. Acute rejection occurs in over 80% of hand and face transplant recipients, significantly higher than in solid organ transplantation, and most often occurs within the first year (78). Costimulation blockade, siRNA therapy, and phototherapy have been novel strategies in immunomodulation that may allow for more targeted and less toxic immunosuppressive regimens (3, 5, 6). However, these strategies have roots in solid organ transplantation and may not translate directly to VCA due to factors such as the unique antigenicity of different tissues of the composite graft.

Local immunosuppression methods have spanned topical treatments, *in situ* implants, and intra-graft injections, which have achieved high local drug concentrations, reduced systemic exposure, and prolonged graft survival in preclinical and early clinical models (8–16, 18). Most recently in 2025, controlled, long-term release depots have been developed that feature burst suppression and reversibility functionalities to significantly improve survival in rat VCA models (17). However, there are still challenges in controlling drug release, tissue concentrations, and systemic immunosuppression adjuncts, reflected in the lack of robust clinical translation studies currently available.

Stem cell therapies can induce tolerance and tissue regeneration, though their use in VCA has been primarily in tolerance induction. Innovations in tolerance induction have converged toward mechanisms that expand and activate regulatory T cells, from dendritic cell education to CCL22-recruitment microparticles (25) and immunomodulation with MSCs (33). Recipient dendritic cells pulsed with donor allopeptide as well as TIM-3-modified dendritic cells have both been shown to prolong VCA survival; however, the findings from these studies are limited by a moderate to high risk of bias from low study sample size and incomplete outcome reporting and statistics (22, 24). In rat VCA models, MSCs led to stable chimerism (14/15 recipients vs. 2/7 controls), boosted Treg populations, and reduced arterial intimal thickness, whereas adipose-derived stem cells with modified CCR7 targeting were able to modulate immune responses within secondary lymphoid organs (32, 33). Further innovations involving the regenerative potential of stem cells could be applied to nerve, muscle, and vascular repair for functional recovery after VCA. The creation of a large scale, cryopreserved bone marrow bank in 2021 overcame the issue of cell yield (35), though implementing such a bank clinically would require extensive infrastructure resources likely beyond what any single center could achieve.

However, there are limitations to translation of such therapies because of the need for toxic conditioning (32), adjunct immunosuppression (27), and the lack of robust human clinical data (35). Research has attempted to balance the success of inducing tolerance with avoiding immune rejection of the therapeutic cells themselves by exploring sources such as donor-derived cells (26), recipient-derived cells (22, 27), and more recently, chimeric fusion approaches (28), even with multiple unrelated donors (29). Therefore, it is likely that stem cell approaches will be adjuncts to standard VCA immunosuppression rather than replacements for the near future, though they remain promising for continued exploration.

Nerve regeneration, and by extension, functional recovery after VCA, are relatively underdeveloped frontiers in innovation. While many studies have attempted to better characterize the underlying neurobiology of nerve regeneration, few technological solutions have been deployed in this space. The intersection of immunosuppression and nerve regeneration is a promising direction for further study. For example, although tacrolimus alone is associated with neurotoxicity, triple immunosuppressive therapy has been shown to enhance electrophysiologic recovery (39). Perhaps these findings suggest that functional restoration and rejection prevention could one day be synergistic.

Novel VCA surgical techniques such as lymphatic modulation have improved surgical reliability, while crossmatch testing and haplotyping have enhanced compatibility in large animal models, facilitating translational research (42, 79). Polyethylene glycol fusion is being explored to improve graft survival and accelerate functional recovery (41).

Digital innovations, including 3D modeling and virtual surgical planning, enhance VCA precision through detailed segmentation and reconstruction, custom cutting guide design, and preoperative simulation for practice, as well as intraoperative guidance that reduces operative time, minimizes technical errors, and enhances graft placement accuracy (45–52, 54, 55). Nevertheless, the use of digital innovations for post-transplant monitoring is currently limited. Early-stage point of care tacrolimus detection devices in VCA lack sufficient clinical validation (55). The field has invested heavily to make digital surgical planning precise but has not built equivalent systems for post-operative monitoring. Given that rejection occurs dynamically, early detection can prevent irreversible damage, and immunosuppression could be personally tailored with more consistent monitoring. Solid organ transplant research has incorporated strategies such as wearable sensors to detect changes in vital signs or inflammatory markers and AI-driven prediction models for rejection that could be explored further in VCA (80). Integration with digital health platforms for longitudinal monitoring could also be considered with an eye to using these innovations to expand equitable access to VCA.

VCA graft preservation has advanced beyond static cold storage through ex situ machine perfusion and subzero nonfreezing supercooling, which demonstrate superior tissue viability and extended preservation times in preclinical models (44, 58–63). However, limitations include perfusion-induced edema, cryoprotectant cytotoxicity, technical complexity, and lack of large animal or human validation. Thus, more needs to be done in optimizing perfusion parameters, integrating real-time viability assessment, minimizing reperfusion injury, and exploring functional outcomes in clinical models.

Tissue-specific biomarker identification shows promise for predicting VCA rejection, but current challenges involve heterogeneous tissue types, surgical injury confounding rejection signals, and lack of consensus on sampling protocols (64, 65). In an attempt to facilitate easier and quicker monitoring of tacrolimus levels in VCA, salivary testing was attempted but showed only weak correlation to serum tacrolimus levels with a high degree of error that is clinically relevant given the drug's narrow therapeutic window (56). Researchers in solid organ transplantation have explored alternative approaches to immunosuppression monitoring, such as dried blood spot testing and volumetric absorptive microsampling. Volumetric absorptive microsampling has been shown in 20 human kidney and liver transplant recipients to have a strong correlation (r = 0.93) with venous tacrolimus levels (81). Such methods of microsampling have been preliminarily investigated in VCA, such as by Lantigua et al. (55) who developed a paper-based device to detect and broadly categorize tacrolimus levels through colorimetric detection. In its current state, the colorimetric detection is too coarse to distinguish between therapeutically meaningful doses of tacrolimus, preventing clinical translation. The study did not address how the device performed compared to venous tacrolimus levels or what drug levels were across multiple tissue types. Application of other microarray techniques such as volumetric sampling or dried blood spot testing to VCA is complicated by tissue-specific immune responses that make it unclear still if systemic tacrolimus levels are an accurate reflection of drug activity within distinct tissues. Even so, further exploration could yield important insights into quicker and less-invasive monitoring of immunosuppression in VCA. Kauke et al. have addressed some of these challenges by using CT scans to quantify axillary sinus aeration coefficient, thereby creating a potential non-invasive rejection marker that allows an earlier indication of rejection in deeper tissues, a limitation of skin biopsies (67). Research into standardized sample collection for tissue type and timing, biomarker-driven clinical decision support, and real-time monitoring for early rejection may expand the application of these biomarker strategies in the future.

Systems-level innovations in VCA are primarily limited by small sample sizes that limit generalizability. Outcomes could be improved by focusing on psychosocial factors implicated in transplantation success, more donor engagement, and collaborative data sharing.

Overall, there has been much exciting innovation within the field that is converging on common problems like rejection and operative complexity from multiple angles. As these technologies converge, we imagine a VCA environment that streamlines and standardizes how these procedures are conducted and monitored.

Several limitations continue to affect research progress in VCA. First, preclinical rodent models are not a perfect reflection of VCA in humans. Second, in clinical trials, small patient cohorts that reduce statistical power. Third, while being currently worked on, there is limited consensus on outcome measures for VCA success. Some studies emphasize graft survival and histologic evidence of rejection, while others emphasize functional recovery. Greater standardization of outcome measures would promote more consistent reporting.

Nevertheless, countless other technologies have experienced this same evolution through both incremental and transformative innovations (82). Many of the technologies discussed above provide incremental changes but lay the foundation for transformative ideas. For example, while we have been able to gradually improve the effectiveness and reduce the toxicity of drug-based immunosuppression, it will hopefully be an innovation that produces a truly chimeric immune system to allow VCA without the need for lifelong drug therapy. Similarly, while we have incrementally improved our techniques in nerve repair, we continue to be hampered by the speed or nerve growth and the senescence of motor endplates. With ongoing work, we hope to achieve rapid and reliable nerve regeneration. Finally, and no less importantly, these technologies are applicable to many other areas of medicine. We look forward to the time when innovation developed in VCA may help patients with other types of transplants, injuries, or deficits.

## Conclusions

Technological innovation in VCA is pushing the frontiers of immunosuppression delivery, nerve regeneration, stem cell therapies, digital innovations, and complex procedural barriers. Studies show that belatacept, phototherapy, and siRNA are novel immunologic strategies that may reduce systemic immunosuppression requirements (3, 5, 6, 20, 23, 24). Mesenchymal stem cell and growth hormone therapies can enhance graft tolerance and reduce the need for systemic immunosuppression (32–35, 38). Digital methods adopt 3D modeling, virtual surgical planning, and precision monitoring of immunosuppression reduce operative complexity (40, 42, 45–53, 55, 56). Enhanced preservation methods such as subnormothermic machine perfusion and subzero non-freezing supercooling extend graft viability beyond static cold storage (60, 62). Computational proteomics enable individualized monitoring, and noninvasive predictive tools of rejection can enable early and more accessible rejection detection (64–68). Systems-level innovations through standardized protocols, improved multidisciplinary team dynamics, and a focus on patient-reported outcomes further optimize program efficiency and results (69–77). These converging technological advances collectively position VCA for significant clinical advancement.

## Data Availability

The original contributions presented in the study are included in the article/Supplementary Material, further inquiries can be directed to the corresponding author.
