# Supplementary material for: Systematic review of emerging technologies in vascularized composite allotransplantation
Source: Front Transplant. 2026 Mar 9;5:1760147. doi: 10.3389/frtra.2026.1760147 (PMC13006663; doi:10.3389/frtra.2026.1760147)
Supplement: Supplementary file 1 [file Table1.docx]

# Appendix

Appendix 1: Search strategy in PubMed, December 27, 2025

| Query |
| --- |
| ("Vascularized Composite Allotransplantation"[MeSH Terms] OR “Vascularized Composite Allotransplantation”[tiab] OR “Composite Tissue Allotransplantation”[tiab] OR "Hand Transplantation"[MeSH Terms] OR "Extremity Transplantation"[tiab] OR “Face Transplantation”[tiab] OR "VCA"[tiab] OR “CTA”[tiab])  AND  ("Challenges"[tiab] OR "Barriers"[tiab] OR "Obstacles"[tiab] OR "Ethics"[MeSH Terms] OR “Complications”[tiab] OR "Graft Rejection"[MeSH Terms])  AND  (Technolog*[tiab] OR Automat*[tiab] OR "Innovation"[tiab] OR "Development"[tiab] OR "New Techniques"[tiab] OR Therap*[tiab] OR Discover*[tiab] OR "Bioengineering"[MeSH Terms] OR "Tissue Engineering"[MeSH Terms] OR “Breakthrough”[tiab])  AND  English [la] |

Appendix 2: Search strategy in EMBASE, December 27, 2025

| Query |
| --- |
| ('vascularized composite allotransplantation' OR 'vascularized composite allotransplantation':ti,ab OR 'composite tissue allotransplantation':ti,ab OR 'hand transplantation' OR 'extremity transplantation':ti,ab OR 'face transplantation':ti,ab OR 'vca':ti,ab OR 'cta':ti,ab)  AND  ('challenges':ti,ab OR 'barriers':ti,ab OR 'obstacles':ti,ab OR 'ethics' OR 'complications':ti,ab OR 'graft rejection')  AND  ('technolog*':ti,ab OR 'automat*':ti,ab OR 'innovation':ti,ab OR 'development':ti,ab OR 'new techniques':ti,ab OR 'therap*':ti,ab OR 'discover*':ti,ab OR 'bioengineering' OR 'tissue engineering' OR 'breakthrough':ti,ab)  AND 'english':la  AND [article]/lim |

Appendix 3: Risk of Bias Analysis

| **Author** | **Title** | **Study Design** | **Risk of Bias Assessment** |
| --- | --- | --- | --- |
| *Systemic Immunosuppression Strategies* | | | |
| Grahammer et al. | Benefits and limitations of belatacept in 4 hand-transplanted patients | Human case series | Low risk |
| Hui-Chou et al. | Short-Term Application of Doxorubicin Chemotherapy Immunosuppressive Side Effects for Composite Tissue Allotransplantation | Animal intervention study | Low to moderate risk |
| Liu et al. | The Synergistic Effect of Full-Spectrum Light Therapy and Transient Immunosuppressants Prolonged Allotransplant Survival | Animal intervention study | Low to moderate risk |
| Chang et al. | Downregulation of Janus kinase 3 expression by small interfering RNA in rat composite tissue allotransplantation | Animal intervention study | Low to moderate risk |
| Adil et al. | Recellularization of Bioengineered Scaffolds for Vascular Composite Allotransplantation | Narrative | Moderate risk |
| *Targeted Drug Delivery* | | | |
| Gharb et al. | Effectiveness of Topical Immunosuppressants in Prevention and Treatment of Rejection in Face Allotransplantation | Animal intervention study | Low to moderate risk |
| Fries et al. | Graft-implanted, enzyme responsive, tacrolimus-eluting hydrogel enables long-term survival of orthotopic porcine limb vascularized composite allografts: A proof of concept study. | Animal intervention study | Low risk |
| Feturi et al. | Tacrolimus-Eluting Disk within the Allograft Enables Vascularized Composite Allograft Survival with Site-Specific Immunosuppression without Systemic Toxicity | Animal intervention study | Low to moderate risk |
| Feturi et al. | Topical Tacrolimus and Mycophenolic Acid Therapy Synergizes with Low Dose Systemic Immunosuppression to Sustain Vascularized Composite Allograft Survival | Animal intervention study | Low to moderate risk |
| Chen et al. | Topical tacrolimus and steroids modulate T cells in acute rejection of hand allotransplantation: Two case reports. | Human case report series | Low risk |
| Gama et al. | Local Immunosuppression for Vascularized Composite Allografts: Application of Topical FK506-TyroSpheres in a Nonhuman Primate Model. | Animal intervention study | Moderate to high risk |
| Wang et al. | Evaluation of PLGA microspheres with triple regimen on long-term survival of vascularized composite allograft – an experimental study | Animal intervention study | Low risk |
| Olariu et al. | Intra-graft injection of tacrolimus promotes survival of vascularized composite allotransplantation | Animal intervention study | Low risk |
| Arenas Hoyos et al. | A local drug delivery system prolongs graft survival by dampening T cell infiltration and neutrophil extracellular trap formation in vascularized composite allografts | Animal intervention study | Low risk |
| Sutter et al. | Delivery of Rapamycin Using In Situ Forming Implants Promotes Immunoregulation and Vascularized Composite Allograft Survival | Animal intervention study | Low risk |
| Lee et al. | A Precisely Controlled Long-Acting Immunosuppression Platform Enables Prolonged Survival of Vascularized Composite Allografts | Animal intervention study | Low risk |
| *Tolerance Induction* | | | |
| Huang et al. | Greater Efficacy of Tolerance Induction with Cyclosporine versus Tacrolimus in Composite Tissue Allotransplants with Less Myeloablative Conditioning | Animal intervention study | Low risk |
| Lin et al. | Vascularized Osteomyocutaneous Allografts Are Permissive to Tolerance by Induction-Based Immunomodulatory Therapy | Animal intervention study | Low risk |
| Jindal et al. | Spontaneous Resolution of Acute Rejection and Tolerance Induction With IL-2 Fusion Protein in Vascularized Composite Allotransplantation | Animal intervention study | Low risk |
| Nguyen et al. | Allopeptide-pulsed dendritic cells and composite tissue allograft survival | Animal intervention study | Moderate to high risk |
| Kuo et al. | Alloantigen-Pulsed Host Dendritic Cells Induce T-Cell Regulation and Prolong Allograft Survival in a Rat Model of Hindlimb Allotransplantation | Animal intervention study | Low risk |
| Wang et al. | The amelioration of composite tissue allograft rejection by TIM-3-modified dendritic cell: Regulation of the balance of regulatory and effector T cells | Animal intervention study | Moderate to high risk |
| Fisher et al. | In situ recruitment of regulatory T cells promotes donor-specific tolerance in vascularized composite allotransplantation | Animal intervention study | Low risk |
| Radu et al. | Donor-derived transplant acceptance-inducing cells in composite tissue allotransplantation | Animal intervention study | Moderate risk |
| Schweizer et al. | Adipose-derived stromal cell therapy combined with a short course nonmyeloablative conditioning promotes long-term graft tolerance in vascularized composite allotransplantation | Animal intervention study | Moderate risk |
| Cwykiel et al. | Donor Recipient Chimeric Cells Induce Chimerism and Extend Survival of Vascularized Composite Allografts | Animal intervention study | Low risk |
| Siemionowet al. | Creation of human hematopoietic chimeric cell (HHCC) line as a novel strategy for tolerance induction in transplantation | *in vitro* experimental study | Low to moderate risk |
| Xu et al. | Simultaneous Bone Marrow and Composite Tissue Transplantation in Rats Treated With Nonmyeloablative Conditioning Promotes Tolerance | Animal intervention study | Low risk |
| Lin et al. | The intragraft vascularized bone marrow induces secondary donor-specific mystacial pad allograft tolerance | Animal intervention study | Low risk |
| *Stem Cell Therapies* | | | |
| Pan et al. | Mesenchymal Stem Cells Enhance the Induction of Mixed Chimerism and Tolerance to Rat Hind-Limb Allografts after Bone Marrow Transplantation | Animal intervention study | Moderate risk |
| Plock et al. | The Influence of Timing and Frequency of Adipose-Derived Mesenchymal Stem Cell Therapy on Immunomodulation Outcomes After Vascularized Composite Allotransplantation | Animal intervention study | Low to moderate risk |
| Soares et al. | Ex vivo allotransplantation engineering: Delivery of mesenchymal stem cells prolongs rejection-free allograft survival | Animal intervention study | Low to moderate risk |
| Johnstone et al. | A Large-Scale Bank of Organ Donor Bone Marrow and Matched Mesenchymal Stem Cells for Promoting Immunomodulation and Transplant Tolerance | Narrative | Moderate quality |
| Chang et al. | Long-term Tolerance Toward Haploidentical Vascularized Composite Allograft Transplantation in a Canine Model Using Bone Marrow or Mobilized Stem Cells | Animal intervention study | Low to moderate risk |
| Ma et al. | Targeted Migration of Human Adipose-Derived Stem Cells to Secondary Lymphoid Organs Enhances Their Immunomodulatory Effect and Prolongs the Survival of Allografted Vascularized Composites | Animal intervention study | Low to moderate risk |
| *Nerve Regeneration and Functional Recovery* | | | |
| Rath et al. | The Effects of Growth Hormone on Nerve Regeneration and Alloimmunity in Vascularized Composite Allotransplantation | Animal intervention study | Low risk |
| Kim et al. | The effect of full dose composite tissue allotransplantation immunosuppression on allograft motor nerve regeneration in a rat sciatic nerve model | Animal intervention study | Low risk |
| *Advanced Surgical Techniques* | | | |
| Kim et al. | Improved Cuff Technique and Intraoperative Detection of Vascular Complications for Hind Limb Transplantation in Mice | Animal intervention study | Moderate to high risk |
| Zhang et al. | A novel strategy for spinal cord reconstruction via vascularized allogeneic spinal cord transplantation combine spinal cord fusion | Animal intervention study | Low risk |
| Pei et al. | Inhibition of Lymphatic Drainage With a Self-Designed Surgical Approach Prolongs the Vascularized Skin Allograft Survival in Rats | Animal intervention study | Low risk |
| Larsen et al. | Living bone allotransplants survive by surgical angiogenesis alone: development of a novel method of composite tissue allotransplantation | Animal intervention study | Low risk |
| Nguyen et al. | Face transplant perfusion assessment using near-infrared fluorescence imaging | Animal intervention study | Moderate to high risk |
| *Digital Innovation* | | | |
| Cornelius et al. | Iterations of computer- and template assisted mandibular or maxillary reconstruction with free flaps containing the lateral scapular border – Evolution of a biplanar plug-on cutting guide | Human case series | Low risk |
| Antony et al. | Use of Virtual Surgery and Stereolithography-Guided Osteotomy for Mandibular Reconstruction with the Free Fibula | Human case series | Low to moderate risk |
| Ciocca et al. | The Design and Rapid Prototyping of Surgical Guides and Bone Plates to Support Iliac Free Flaps for Mandible Reconstruction | Narrative | Moderate risk |
| Jacobs et al. | Best Face Forward: Virtual Modeling and Custom Device Fabrication to Optimize Craniofacial Vascularized Composite Allotransplantation | Narrative | Low risk |
| Vyas et al. | Virtual Surgical Planning and 3D-Printed Surgical Guides in Facial Allotransplantation | Narrative | Low risk |
| Soga et al. | Noninvasive vascular images for face transplant surgical planning | Human case series | Low risk |
| Cho et al. | Mixed Reality and 3D Printed Models for Planning and Execution of Face Transplantation | Narrative | Low to moderate risk |
| Murphy et al. | Optimizing Hybrid Occlusion in Face-Jaw-Teeth Transplantation: A Preliminary Assessment of Real-Time Cephalometry as Part of the Computer-Assisted Planning and Execution Workstation for Craniomaxillofacial Surgery | Proof-of-concept | Low to moderate risk |
| Coombs et al. | Skeletal and Dental Outcomes after Facial Allotransplantation: The Cleveland Clinic Experience and Systematic Review of the Literature | Human case series | Low to moderate risk |
| Shah et al. | Computerized Surgical Planning in Face Transplantation | Human case series | Low to moderate risk |
| Lantigua et al. | A new paper-based biosensor for therapeutic drug monitoring | Proof-of-concept | Low risk |
| Charlès et al. | Immunosuppressant drug monitor: A non-invasive device to measure tacrolimus level in the saliva of transplanted patients | Diagnostic Test Accuracy Study | Moderate risk |
| *Graft Preservation and Perfusion* | | | |
| Chakradhar et al. | Ischemia Time in Extremity Allotransplantation: A Comprehensive Review | Narrative | Low risk |
| Ward et al. | The role of nitric oxide synthase and heme oxygenase in the protective effect of hypothermia in ischemia-reperfusion injury | Animal intervention study | Low to moderate risk |
| Werner et al. | Ex Situ Perfusion of Human Limb Allografts for 24 Hours | Human case series | Low risk |
| Goutard et al. | Exceeding the Limits of Static Cold Storage in Limb Transplantation Using Subnormothermic Machine Perfusion | Animal intervention study | Low risk |
| Goutard et al. | Machine Perfusion Enables 24-h Preservation of Vascularized Composite Allografts in a Swine Model of Allotransplantation | Animal intervention study | Low risk |
| Filz Von Reiterdank et al. | Supercooling preservation of vascularized composite allografts through CPA optimization, thermal tracking, and stepwise loading techniques | Ex vivo animal study | Low risk |
| Filz Von Reiterdank et al. | Enhanced VCA Storage: A Pilot Study Demonstrating Supercooling in Orthotopic Rodent Hindlimb Transplantation | Proof-of-concept | Low to moderate risk |
| *Biomarkers* | | | |
| Wolfram et al. | Insights from computational modeling in inflammation and acute rejection in limb transplantation | Animal intervention + computational modeling | Low risk |
| Kuo et al. | Proteomic analysis in serum of rat hind-limb allograft tolerance induced by immunosuppressive therapy with adipose-derived stem cells | Animal intervention study | Moderate risk |
| Zor et al. | Reflectance confocal microscopy as a useful diagnostic tool for monitoring of skin containing vascularized composite allograft rejection: A preliminary study on rats | Diagnostic Test Accuracy Study in Animal Model | Low to moderate risk |
| Kauke et al. | Dynamic Maxillary Sinus Changes of Facial Vascularized Composite Allotransplants | Human case series | Low risk |
| *Systems-level Innovations* | | | |
| Siemionow et al. | Overview of guidelines for establishing a face transplant program: a work in progress | Program development report | Low risk |
| Tyner et al. | Measuring Health-Related Quality of Life in Upper Extremity Vascularized Composite Allotransplantation: Development of New Patient-Reported Outcome Items for Hand Transplant | Qualitative | Low risk |
| Sweeney et al. | Perioperative Nursing Management of Donor and Recipient Patients Undergoing Face Transplantation | Program development report | Low risk |
| Griffin et al. | Using team science in vascularized composite allotransplantation to improve team and patient outcomes | Qualitative case study | Low risk |
| Siminoff et al. | Developing online communication training to request donation for vascularized composite allotransplantation (VCA): improving performance to match new US organ donation targets | Program development report | Low risk |
| Kinsley et al. | A Provider Perspective of Psychosocial Predictors of Upper-Extremity Vascularized Composite Allotransplantation Success | Qualitative | Low risk |
| Tyner et al. | Assessment of quality of life after upper extremity transplantation: Framework for patient-reported outcome scale domains | Framework Development | Low risk |
